# DISMIRA: Prioritization of disease candidates in miRNA-disease associations based on maximum weighted matching inference model and motif-based analysis

**DOI:** 10.1186/1471-2164-16-S5-S12

**Published:** 2015-05-26

**Authors:** Joseph J Nalluri, Bhanu K Kamapantula, Debmalya Barh, Neha Jain, Antaripa Bhattacharya, Sintia Silva de Almeida, Rommel Thiago Juca Ramos, Artur Silva, Vasco Azevedo, Preetam Ghosh

**Affiliations:** 1 Department of Computer Science, Virginia Commonwealth University, 401 W Main St, Richmond, VA, USA; 2 Centre for Genomics and Applied Gene Technology, Institute of Integrative Omics and Applied Biotechnology (IIOAB), Nonakuri, Purba Medinipur, 721172 West Bengal, India; 3 Laboratorio de Genetica Celular eMolecular, Departmento de Biologia Geral, Instituto de Ciencias Biologic Belo Horizonte, Minas Gerais, Brazil, Universidade Federal de Minas Gerais CP 486, CEP 31270-901 Minas Gerais, Brazil; 4 Instituto de Ciências Biológicas, Universidade Federal do Pará, Belém, PA, Brazil; 5 School of Biotechnology, Devi Ahilya University, Indore, India

**Keywords:** miRNA-disease regulation, graph theory, network optimization, motifs

## Abstract

**Background:**

MicroRNAs (miRNAs) have increasingly been found to regulate diseases at a significant level. The interaction of miRNA and diseases is a complex web of multilevel interactions, given the fact that a miRNA regulates upto 50 or more diseases and miRNAs/diseases work in clusters. The clear patterns of miRNA regulations in a disease are still elusive.

**Methods:**

In this work, we approach the miRNA-disease interactions from a network scientific perspective and devise two approaches - maximum weighted matching model (a graph theoretical algorithm which provides the result by solving an optimization equation of selecting the most prominent set of diseases) and motif-based analyses (which investigates the motifs of the miRNA-disease network and selects the most prominent set of diseases based on their maximum number of participation in motifs, thereby revealing the miRNA-disease interaction dynamics) to determine and prioritize the set of diseases which are most certainly impacted upon the activation of a group of queried miRNAs, in a miRNA-disease network.

**Results and Conclusion:**

Our tool, *DISMIRA *implements the above mentioned approaches and presents an interactive visualization which helps the user in exploring the networking dynamics of miRNAs and diseases by analyzing their neighbors, paths and topological features. A set of miRNAs can be used in this analysis to get the associated diseases for the input group of miRs with ranks and also further analysis can be done to find key miRs or diseases, shortest paths etc. *DISMIRA *can be accessed online for free at http://bnet.egr.vcu.edu:8080/dismira.

## Background

MicroRNAs are small length (~22nt) non-coding RNAs that inhibit the expression of a target mRNA by binding to its 3'-UTR through complimentary base pairing [1] and therefore, these miRNAs act as negative regulators of the gene expression [2-4]. A mature miRNA regulates the post transcriptional gene expression by targeting certain mRNAs, subsequent to which, it modulates multiple signaling pathways, biological processes and patho-physiologies. However, it has also been evidenced that in some cases, miRNAs act as positive regulators of gene expression [5,6]. Hence, analysis and in-depth exploration of the precise mechanism through which the regulatory mechanism of miRNA exerts its functionality is crucial. Identifying and predicting miRNA and disease associations, has been extensively researched in the past few years [7-10]. However, the precise mechanisms of miRNAs regulating diseases are still unclear. A major portion of the problem persists because about 60% of the molecular bases of diseases are yet unknown [11]. Furthermore, models to predict or determine disease-miRNA associations with high accuracy are very few [12]. Hence, gathering valuable evidence regarding identification of miRNAs influencing human diseases has become a widespread interest in arena of biomedical research with a future looking towards the enhancement of human medicine [13]. In this paper, we investigate the miRNA-disease network from a graph theoretical perspective and devise network scientific models of maximum weighted matching and motif-based analyses, to prioritize disease candidates in a miRNA-disease network. This work also presents a tool, *DISMIRA *that can perform these analyses and display the network visualization of the results, thereby providing an insight into the nature of networking between miRNAs and their associated diseases.

### miRNA disease database - *miRegulome*

To facilitate this, an in-house database, *miRegulome *(freely available at [14]) has been created. This database provides substantial details about the entire regulatory modules of a miRNA curated from PubMed indexed literature. It contains the upstream regulators and chemicals which regulate a miRNA, the downstream targets of a miRNA, miRNA-regulated pathways, functions and diseases along with their associated PubMed IDs. Currently, miRegulome contains information pertaining to 613 miRNAs, 156 diseases, 305 pathways and 96 chemicals. This data has been curated from 3298 PubMed IDs. *miRegulome *currently has 3751 unique miRNA- disease associations with supporting PubMed IDs.

The work presented in this research uses the data gathered in *miRegulome *database.

### Complex networks

Identification of miRNA-disease associations through experimental laboratory methods are time consuming and expensive [7]. Hence, a large interest has been devoted towards finding important underlying associations through various computational models.

**Figure 1:**
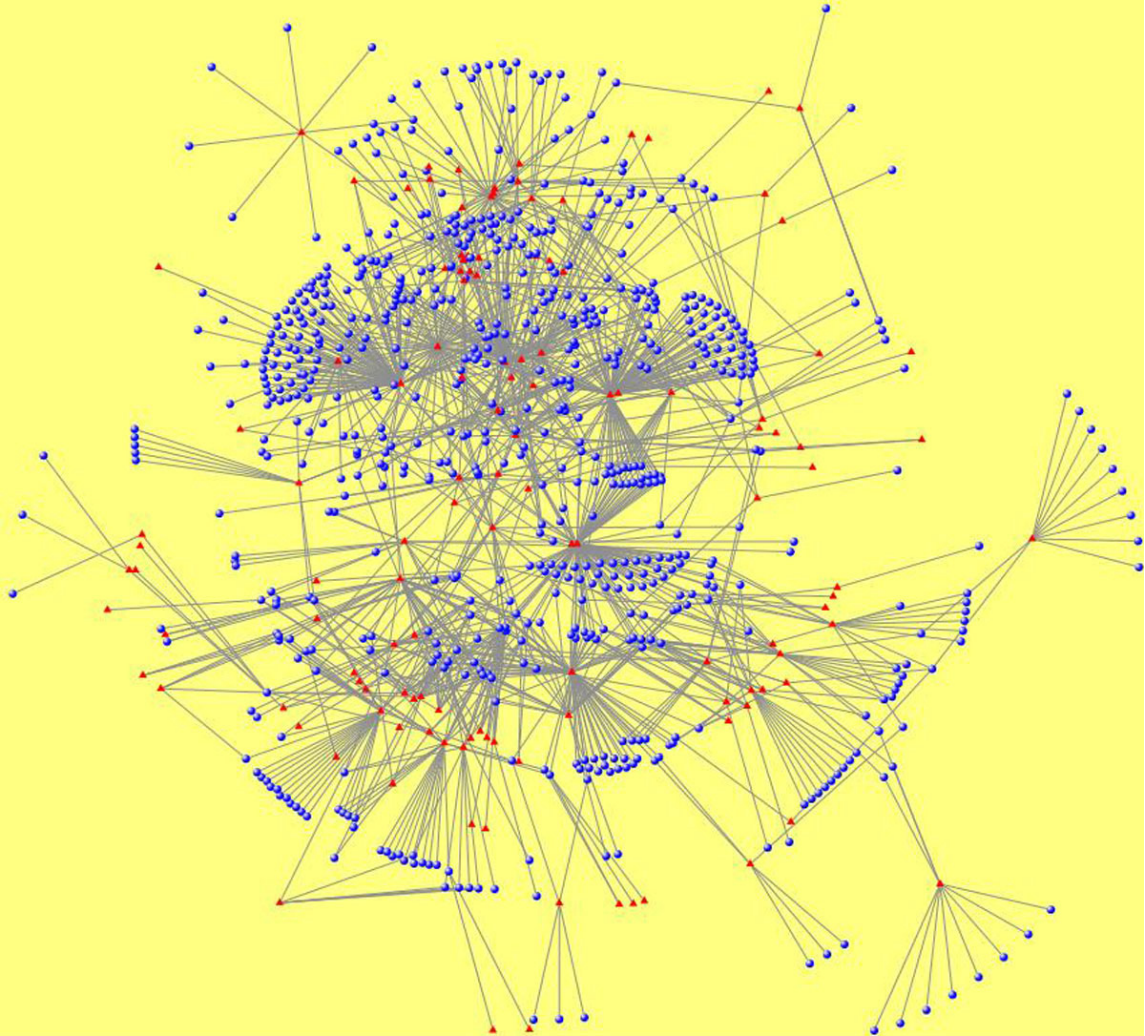
**Network of miRNA-disease associations in HMDD**. Blue circles represent miRNAs and red triangles represent diseases.

A network of miRNAs and diseases underlain with TFs and target genes is a very dense network and thereby poses a very complex network problem. Complex networks offer a unique perspective to explore relationships among homogeneous and heterogeneous entities. These entities can be biological molecules, diseases, genes etc. Hence, graph theoretic concept is very apt to model and mine important miRNA-disease associations. In our research, almost all the observed miRNA- disease networks, such as *miRegulome, mir2Disease *[15], miRNA-disease association network (MDAN) [1] and Human MicroRNA Disease Database (HMDD) [16] are scale-free; meaning few nodes i.e miRNAs have the highest impact on other nodes, thereby acting as hubs. Hence, a miRNA-disease network follows the topological characteristics of scale-free networks. For e.g. Figure 1 shows a scale free network of miRNA-disease association network of HMDD. Further details about the topological metrics of the scale-free nature of these miRNA-disease networks are elaborated in the Section *Motif-Based Analysis*.

### Literature

There have been many approaches to predict and determine associations between miRNAs and diseases. One of such preliminary works in developing miRNA-disease prediction models demonstrates that miRNAs related to same diseases tend to work together as miRNA groups [15]. This is an significant observation. It necessitates that any model of miRNA-disease association/prediction which claims to be effective considers this dynamic nature of miRNA. Jiang, et al., 2010 [9] uses the same approach and further derives a functional similarity between disease-related miR- NAs and phenotype similarities to derive a score which evaluates the likelihood of association of a miRNA and the disease. Jiang, et al., 2010 [17] uses the disease-gene associations to develop a *N aïve − Bayes *model, which prioritizes candidate miRNAs based on their genomic distribution. This model relies heavily on the associations between gene-disease and interactions of miRNA and target. However, both these models have high false-positives and high false-negatives in their predictions [1]. This limitation was however, addressed [7], by training a support vector machine classifier based on the input set of features extracted from false-positives and false-negative predicted associations. As demonstrated by Lu, et al., 2008 [16], miRNA-set families tend to closely work towards certain diseases. Hence, implicitly diseases tend to affect the working of other diseases too. This has also been researched [18], where specifically prostate cancer and non-prostate cancer miRNAs are distinguished by the usage of topological features. Here, a prioritization of disease candidate was performed using a network-centric method. Apart from using disease-gene information, few models have used the assumption that miRNA loci and Online Mendelian Inheritance in Man (OMIM) disease loci may contain significant overlaps [19]. This significance score is calculated and used to identify potential associations between miRNAs and OMIM diseases. Chen, et al., 2012 [1] uses global network similarity measure as compared to local network information to implement a random walk on a functionally similar miRNA network, which prioritizes candidate miRNAs for specified diseases. Xuan, et al., 2013 [8] improvises the miRNA functionality estimated approach by appending disease phenotype similarity information and content of disease terms to the existing method. This is used to assign weight to miRNA-disease associations and a weighted *k*-most similar neighbor based prediction method is deployed. Global network similarity is also used in the inference methods presented [10], where apart from miRNA-similarity and phenotype-similarity inferences, a network based inference model is used. In this model [10], the miRNAs related to queried miRNA are ranked and associated with ranked disease phenotypes associated with target phenotype, thereby relying on known gene-phenotype associations. Graph theory has been extensively used to model and analyze such biological networks [16] and especially bipartite graph modeling has been used to model the miRNA-disease network [1,10,12,16]. Recently, Chen, et al., 2014 [20] has tried to overcome the limitations posed through various previous works, by developing an algorithm of Regularized Least Squares for miRNA-disease association (RLSMDA). Previous models like that of Chen, et al., 2012 [1] which although demonstrate high accuracy in prediction based on their case studies and cross-validation, cannot work in scenarios where associations between the diseases and miRNAs are unknownn; and hence cannot predict novel miRNA- disease associations. Chen and Zhang [10] addressed this in their work, which could predict novel associations between diseases and miRNAs, with no prior knowledge of their association. However, its performance was inferior to that of Chen, et al. [1] based on cross-validation results [20]. The work presented by Chen, et al. [20] uses the miRNA functional similarity and disease functional similarity [21] and devises an optimization formulation to generate a continuous classification function which calculates the probablity score of each miRNA to a given disease [20]. Using graph theory, some network inference based prediction algorithms have also been used, as in [22]. In this case, three networks: environmental factors (EF)-miRNA, EF-disease and miRNA-disease were modeled into bipartite networks and three methods, i.e. network based inference (NFI) algorithm [23], EF structure similarity- based inference model and disease phenotype similarity-based inference models were was used to generate an EF-miRNA-disease association model which is validated via 10-fold cross validation. The cases studies presented display impressive results. However, this work too, can predict associations between EF-miRNA-disease which are known in prior and does not predict novel associations [22]. Our work does not present miRNA-disease predictions, rather performs a maximum matching in a set of miRNAs and diseases to determine and prioritize diseases with highest cumulative impact. Hence, the resulting diseases, each of them have valid PubMed literature supporting it, and thereby accurate association with miRNAs. This gives the user complete confidence in the results, he/she is provided with. Further more, all other previous tools are prediction models, predicting *a *miRNA-disease edge/association. These models do not produce associations between set of miRNAs onto a set of diseases, thereby not exploring the overall dynamics of multi-level interaction of a miRNA-disease network. Our model which acts as an extension to the existing body of work in this field, works on a set of miRNAs and produces an output of a set of associated diseases, taking into account the impact and association of every miRNA in the set with every disease in the set.

Using the graph theoretical network model, in this work we aim to find the most impacted diseases upon action/altercation of specified miRNAs. Here, we present a model that determines a prioritized set of diseases which are most definitely influenced upon the cumulative action/altercation of specified miRNAs. These associations are determined by a pipeline process of applying the maximum-weighted- maximum-matching algorithm to the network model in Section *Maximum Weighted Matching Inference model*, calculating cumulative weights per disease in Section *Prioritization of disease candidates*, and applying the disease ranking scheme in Section *Disease ranking scheme*. A preliminary version of this work has been presented [24]. Furthermore, none of the previous work have presented any work on the motif analysis of miRNA-disease networks. In this paper, we analyze the topological features of several miRNA-disease networks, especially the motifs in these networks and also the cumulative impact of a set of miRNAs onto a set of diseases. The motif-based analyses is presented in Section *Motif-Based Analysis*. The visualization of these results and their topological perspective is elaborated in the Section *Visualization*.

## Methods

**Figure 2:**
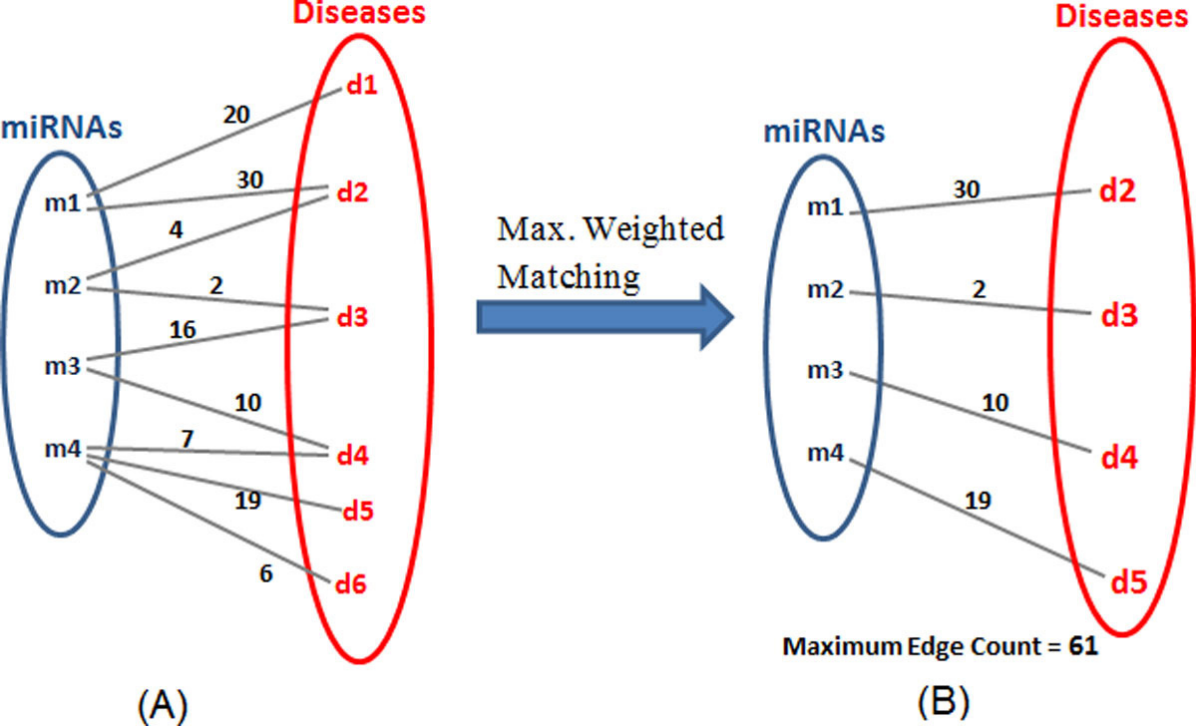
**Maximum weighted matching**.

Single or multiple miRNA(s) is/are up- or down- regulated in one or a set of disease(s). The instances of up and down-regulations between a miRNA and disease, signify the strength of association between the pair. The interactions of miRNAs and diseases can be mapped as a complex network such that miRNAs and diseases are nodes in the network [Figure 1]. This mapping is critical to explore the associations and depends heavily on the type of interactions. A graph theory concept such as bipartite graph [25] can be used to model this problem. In this work, we have modeled the miRNA-disease interaction as a bipartite graph which is shown in Figure 2-A.

### Maximum Weighted Matching Inference model

A bipartite graph is a graph *G*(*V, E*) in which the set of vertices *V *can be partitioned into two disjoint sets *V*1 and *V*2 such that every edge connects a vertex in *V*1 to the one in *V*2 [25]. In our model, miRNAs and diseases have been categorized as two disjoint sets and an edge represents an association between them. The data consisting of miRNAs and diseases has been used from *miRegulome*. Herein, the edges are weighted i.e. the number of publications citing up/down regulations between a miRNA-disease pair. For e.g. in Figure 2-A the edge weight of 20 between *m*1 and *d*1 represents the number of PubMed IDs citing miRNA *m*1 regulating disease *d*1. Hence, the weight of the edge represents the strength of the association between the miRNA and disease. Based on this data, we derive a weighted network consisting of miRNA-disease interactions.

*Maximum weighted matching (MWM): *In the graph *G*(*V, E*), if there is a set of edges such that no two edges share a common end vertex, it is known as a matching. Maximum matching is a matching with largest possible set of edges. A maximum weighted matching is a maximum matching such that the sum of the weights of the edges is maximum. This is explained below.

Consider a miRNA-disease interaction network as in Figure 2, where *m*1 to *m*4 are miRNAs and *d*1 to *d*7 are diseases. The weight on the edge represents the strength of the association between the miRNA-disease pair, in terms of the number of publications citing up-regulating and down-regulating a disease. For e.g. in Figure 2, the edge *m*1-*d*2 has a weight of 30, which indicates there are 30 publications (i.e. PubMed IDs) in the curated literature of *miRegulome *which cite the miRNA *m*1 either up-regulating or down-regulating disease *d*2. As Figure 2-(B), shows after the application of the MWM algorithm, the resultant sum of edges is the maximum score, which implies that there is no possible *combination *of *m*-*d *pairs in the network, whose cumulative sum is higher than the result. Hence, the MWM helps in determining the strongest miRNA-disease pairs combination among a set of active miRNAs. The results give the cumulative impact of a set of activated miRNAs on the set of associated diseases, which are most certainly impacted. The goal is to present a concise list of diseases with highest confidence of being influenced and not to determine specific miRNA-disease associations; rather an association between a *set *of miRNAs onto a *set *of diseases. Models of such association that calculate the cumulative impact of a set miRNAs onto a set of diseases are not many. This is important because miRNAs and diseases tend to interact closely in sets and groups and hence a tool in prioritizing disease candidates is helpful in presenting a comprehensive and yet concise list, displaying the cumulative impact of specified miRNAs.

In our premise, since we are exploring the associations among a set of miRNAs onto a set of diseases, it is important to bear in mind that many diseases might be associated, but not all diseases might be significantly relevant to the set of inputted miRNAs. Hence, we have to consider each miRNA's sphere of influence onto diseases, as well as its relevance to other miRNAs' sphere of influence. Herein, the MWM algorithm addresses the issue, by choosing the optimum set of associations (with highest cumulative sum) that the set of miRNAs present. This algorithm takes into account each miRNA's sphere of influence and its strength of influence/association and thereby calculates a set of edges, in consideration with set of miRNAs such that the resultant cumulative influence of the set of miRNAs onto the set of diseases is highest. In other words, just because a certain miRNA-disease edge has not been selected, it does not imply, it is not considered. What it implies is that, it is not important when the entire set is considered. Also, the goal is to produce a concise list and not an entire set of associated diseases. This constraint does well to generate a set which is both representative of every miRNA's sphere of influence as well as determining the highest impacted diseases.

In any given miRNA-disease network, the solution to the MWM algorithm in a given *G*(*V, E*) can be solved as an optimization problem as described by Fang, 2012 [26]. It suggests the following:

*Optimization problem formulation: *Objective: To achieve the maximum sum of weighted edges between miRNA and diseases, subject to constraints that no vertices share the same edge. This helps us in getting the most prominent collection of pairs such that, their cumulative sum is the maximum among all possible combinations. Variables: Let *X_i,j _*be an edge between a miRNA and disease, *W eight_i,j _*be the edge weight between the miRNA-disease pair, *m *and *d *be the set of miRNAs and diseases respectively. Algebraic formulation:

$$\begin{array}{c} Maximize\underset{i,j}{\sum }Weight_{i,j}*X_{i,j}\\ s.t.\underset{j}{\sum }X_{i,j}\leq 1\left(j=1,2,\ldots ,m\right)\\ \underset{i}{\sum }X_{i,j}\leq 1\left(i=1,2,\ldots ,d\right) \end{array}$$

In the above formulation, we are maximizing the cumulative sum of the edges, with the constraints that no miRNA or disease should be repeated. These constraints help in reducing the repetition of common diseases associated with different miRNAs; since miRNAs tend to regulate about 50 to 100 or more diseases based on data in the human microRNA disease database (HMDD)[16] and *miRegulome*. This is important keeping in view that the goal is to present a breadth of diseases *within *the concise list, bearing on the fact that miRNAs tend to work closely in sets.

The above MWM optimization formulation is a linear programming problem and geometrically, its a convex function. The resulting feasible region of solutions is a polyhedron. This linear programming equation is solved using the linear program (LP) solver GLPSOL which uses the simplex method [27].

#### Prioritization of disease candidates

**Figure 3:**
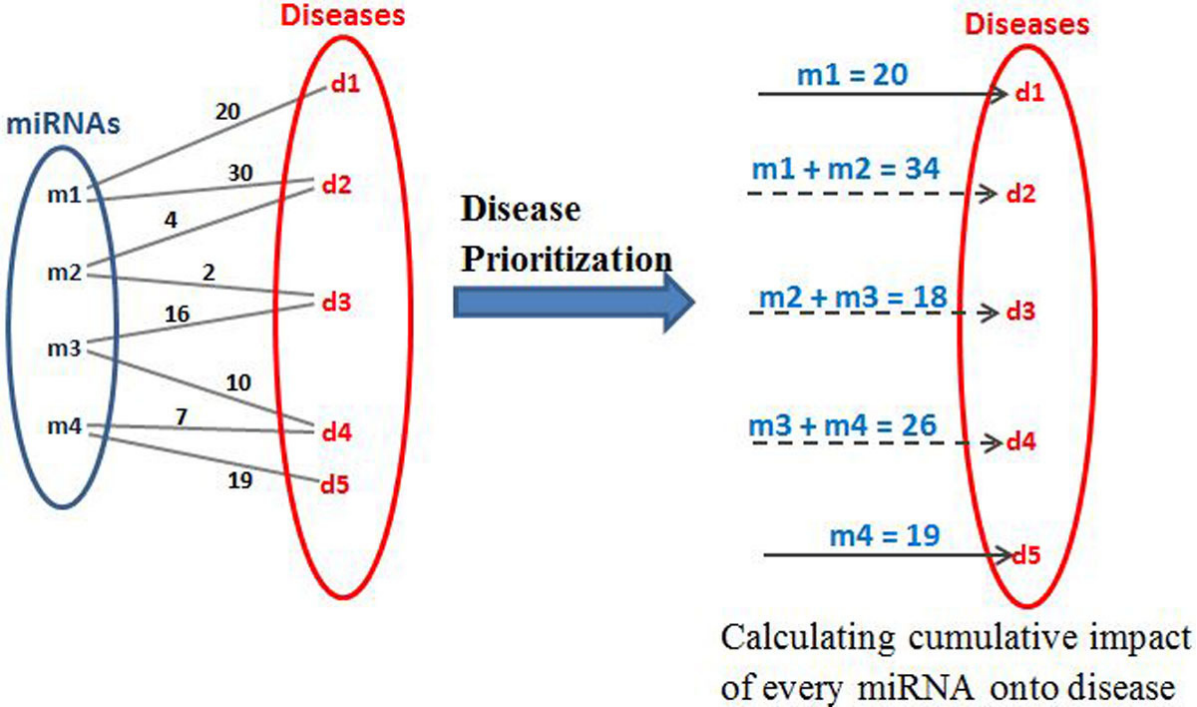
**Cumulative impact of each miRNA**.

Since many miRNAs are connected to a single disease they have a cumulative influence on it. For e.g. in Figure 3, disease *d*2 is influenced by miRNAs *m*1 and *m*2. Similarly, diseases *d*3 and *d*4 are influenced by more than one miRNA. In real scenarios, diseases are regulated by multiple miRNAs. Hence, it is vitally important that we consider the cumulative impact of all the active miRNAs on its associated diseases. In this model after the miRNAs-disease network is created based on user input of active miRNAs, we calculate the cumulative impact of all of them on each connected disease. Figure 3, shows the influence on each diseases numerically. This helps in understanding in many ways, how a disease can be influenced by multiple miRNAs, which is not considered in the MWM model. The MWM model, as shown in Figure 2, selects the top impacted diseases. Each diseases' impact can be calculated by adding the weights of every active miRNA and the particular disease, as shown in Figure 3.

This approach gives a ranked list of diseases.

#### Disease ranking scheme

Although, the application of MWM algorithm gives the most prominent miRNA- disease associations, it has a limitation. Because of the constraint that no two edges can share a common vertex, a strongly associated miRNA-disease pair can get ignored in the MWM selection process. For example, consider miRNAs *m*2 and *m*3 in Figure 2; for miRNA *m*2 and miRNA *m*3, the *m*3 *− d*3 pair weight is 16 and *m*3 *− d*4 pair weight is 10. However, in the resultant matching only *m*3 *− d*4 pair is selected (see Figure 2), because addition of *this *edge provides the highest cumulative sum when all possible resultant combinations are considered. The pairs *m*3 *− d*4 and *m*2 *− d*3 are selected in the matching but their pair weights are 10 and 2 respectively, which is less than the non-selected pair, *m*3 *− d*3. In order to overcome this limitation, a disease ranking scheme has been adopted.

**Figure 4:**
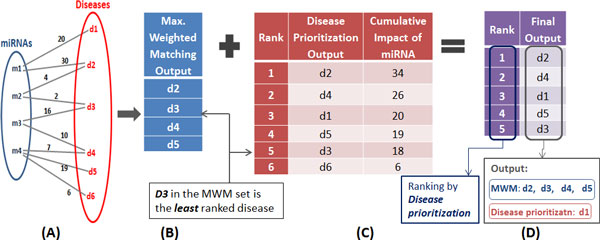
**Complete design of the MWM based model**. **(a) **miRNA-disease set **(b) **Result of MWM algorithm **(c) **Prioritization of diseases as per cumulative impact **(d) **Ranked set of diseases from MWM and **(c)**.

Here, diseases are ranked as per their highest cumulative impact from miRNAs (see Figure 4-C) as explained in Section *Prioritization of disease candidates*. This set of ranked diseases is compared with the set of diseases obtained after the MWM algorithm (See Figure 4-B). The rank of the disease in the MWM set which is least ranked is noted. If there are other diseases which have a higher rank than the least-ranked disease ***and ***are not included in the MWM set; those are added to the final output set of diseases (see Figure 4-D). This method makes sure that a disease which is highly influenced is not missing after the MWM algorithm is applied. MWM algorithm helps in giving a definite and concise set of affected diseases. Prioritizing of diseases ranks them as per their impact. Disease ranking scheme enhances the result set by overcoming the limitation of the MWM algorithm, and adding higher ranked diseases in the final resultant set of diseases.

When the miRNAs are entered by the user, an automated script performs the following functions:

1 Runs the database procedure gathering the relevant literature pertaining to the set of miRNAs

2 Generates the cumulative impact of miRNA onto each disease in a ranked manner (Figure 4-C)

3 Creates a network model of the miRNA-disease associations in GMPL (Figure 4-A)

4 Runs the MWM optimization script which operates on the created network model and generates the optimum set of associations (Figure 4-B)

5 Observes the disease in the results of MWM (Figure 4-B) and identifies the least ranked among them. Thereafter, it checks for diseases with higher cumulative count than the least ranked disease, in the result set of (2), i.e Figure 4-C. If there are any diseases with higher cumulative impact and not included in the MWM set (4), they are added to the resultant set (Figure 4-D) For e.g. in Figure 4, the set of diseases through MWM were *{d2, d3, d4, d5 } *and the set of diseases through 'Disease ranking' were *{d2, d4, d1, d5, d3 }*. Disease *d1 *had higher cumulative impact compared to the least ranked disease *d3 *in the MWM set and hence it was added to the final resultant section. Therefore, the final resultant set of diseases is *{d2, d4, d1, d5, d3 }*

This model has been used on the data from *miRegulome*, HMDD, and *miR2Disease *[15] databases. Table 1 presents some of the results. PubMed IDs are provided for further reference.

**Table 1:** MWM based algorithm results.

**S.No**.	miRNAs	Diseases	PubMeds for results
1	hsa-mir-9-1, hsa-mir-9-2, hsa-mir-200c	Breast cancer, Colorectal cancer, Kidney cancer, Ovarian cancer	23617747

2	hsa-mir-182, hsa-mir-200a, hsa-mir-200b, hsa-mir-200c	Lung cancer, Ovarian cancer (OC), Hepatocellular carcinoma (HCC), Breast cancer, Kidney cancer, Colorectal cancer, Oral squamous cell carcinoma	23272653

3	hsa-mir-29a,	Ulcerative coltis, Serious ovarian cancer, Bladder cancer, Pituary adenoma, Primary Biliary cirrhosis, Epithelial Ovarian Cancer, Cardiac hypertrophy, Breast cancer, Acute Lymphoblastic leukemia, Kidney cancer, Gastric cancer and nasopharyngeal carcinoma	18056805,
	hsa-mir-34a,		19475496,
	hsa-mir-34b,		19646430,
	hsa-mir-25		16461460,
			18728182,
			18390668,
			17823410

This approach stands in contrast with many of the previous approaches mentioned in the *Literature *section. Firstly, most of the previous works, for e.g. [1],[7],[8] and [9] are *prediction *based results and present *'1-to-1' *miRNA-disease association. In contrast, our work explores the associations between the *set *of miRNAs onto *set *of diseases and presents results which are *known *associations, validated by PubMed ids and not predicted. Secondly, our starting premise and motivation for this work, unlike the previous works, is to explore the collaborative working of the sets of miRNAs and diseases. There are not many tools, which determine a set of diseases based on the user's input set of miRNAs to which we can compare. Thirdly, previous works present a list of associations between miRNA and diseases which are static in nature, and predict *new *associations which are valid with certain confidence score. However, the approach and results in our work are dynamic; meaning the results will change everytime a new set of miRNAs are entered. The results are generated at the instant - by *sending a query *to gather the relevant literature, *generating *the network model, *optimizing *the objective of the network model, *calculating *the cumulative impact on diseases and *producing *the set of diseases. As more and more new associations are added to the databases, the results would only change for better. The results are not *new *predictions rather set of *known *diseases, determined and prioritized to the set of input miRNAs. Owing to the aforementioned reasons, there could not be a reasonable and fair comparison done with previously established, benchmarked prediction-based datasets used in [1], [9], [10] and [12] which are static, 1-on-1 miRNA-disease predictions.

### Motif-based analysis

The topological features of miRNA-disease network could provide valuable insights into the nature of collaboration of miRNAs and diseases, since miRNAs emerge to work in groups [16]. It has been observed that motifs are the fundamental building blocks in biological networks [28], since they are frequently occurring substructures. These substructures can be of sizes 2 or above. Hence, we studied the topological features of this network, namely motifs. We performed a motif-based analysis of a miRNA-disease interaction network, and the disease-disease interaction network. *mfinder *[29] and *fanmod *[30] software are used to determine the most significant motifs in the considered miRNA-disease networks. Motifs generated by *mfinder *are identified in green color and motifs generated by *fanmod *are identified in orange color. Apart from the networks derived from *miRegulome*, these motif-based analyses were also performed in *miR2Disease* [15] network and also the HMDD [16] database.

#### mirna-disease network

**Figure 5:**
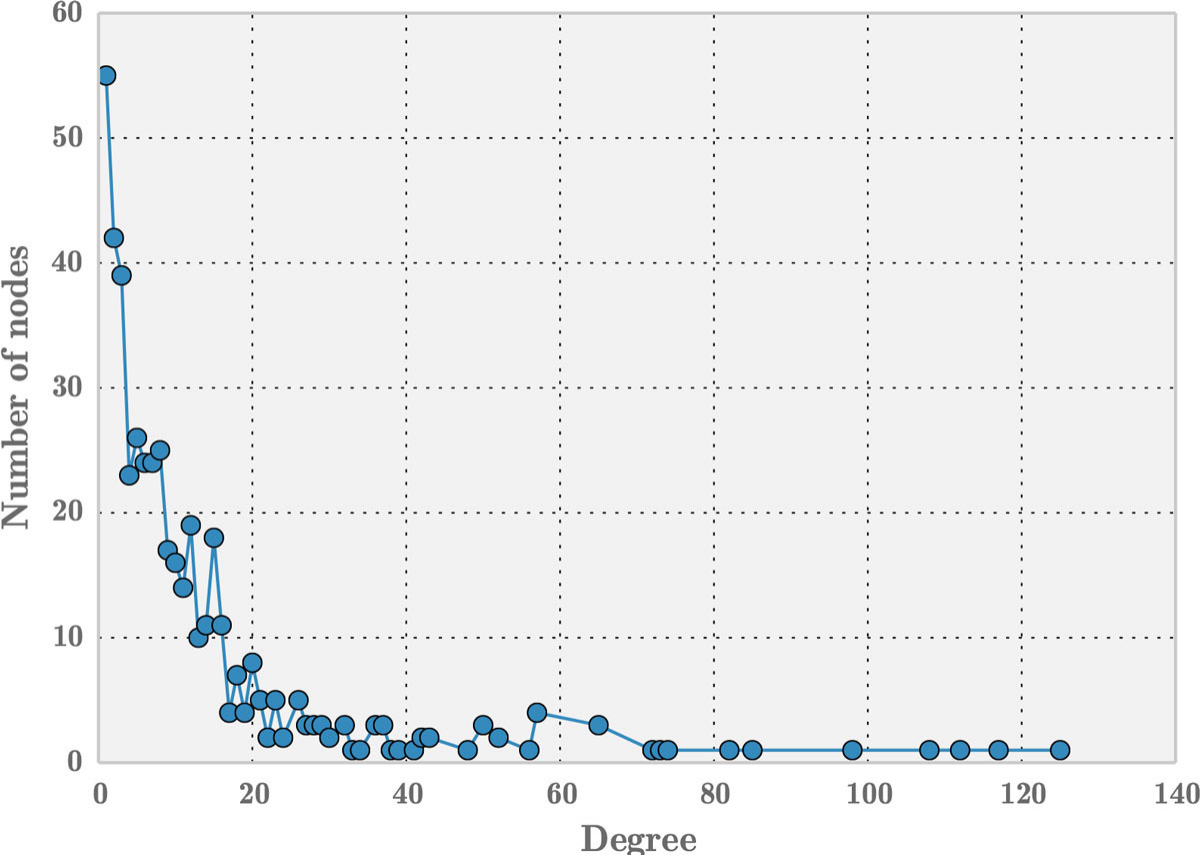
**Degree distribution of miRNA-disease network**.

**Figure 6:**
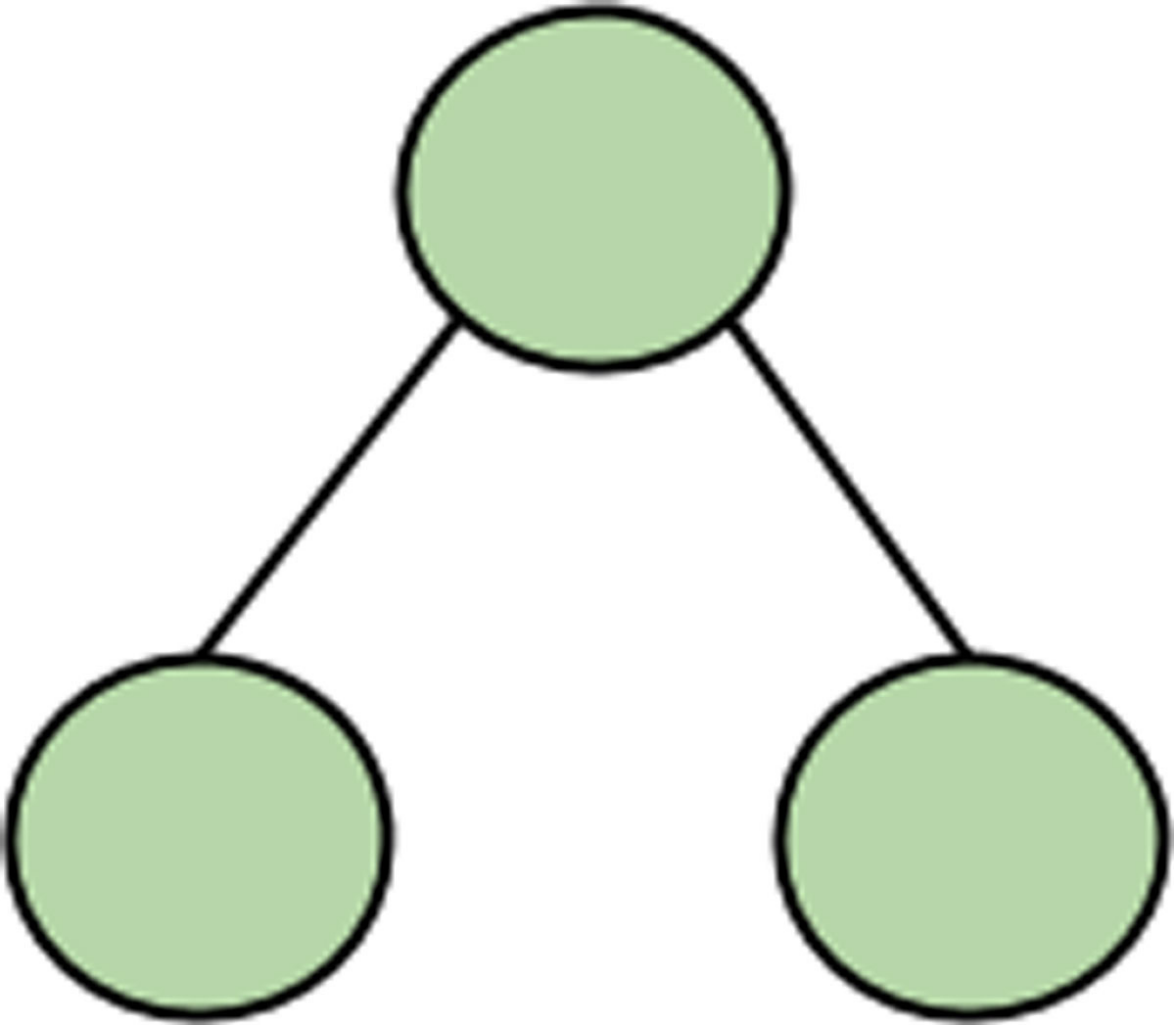
**3 node motif in miRNA-disease network**.

**Figure 7:**
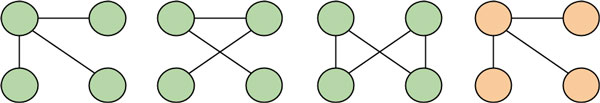
**4 node motifs in miRNA-disease network**.

The miRNA-disease associations obtained from *miRegulome *contained 468 nodes and 2998 edges which is a sparse network with a density value of 0.0273. The degree distribution of miRNA-disease network (see Figure 5) follows power-law property of scale-free networks, i.e. their degree distribution follows the property of *P *(*k*) ~*k−γ *[31]. Earlier research on scale-free networks showed that such networks are modular. While bipartite graph analyses identify diseases that are most influenced by miRNAs using empirical evidence, motif analyses offers an additional perspective by introducing structural insight to the miRNA-disease networks. The following 3 node (see Figure 6) and 4 node motifs (see Figure 7) were found to be significant. The 3 node motif implies there a miRNA regulating atleast two diseases and atleast two miRNAs regulating a single disease. It also corroborates the finding that, when a single disease is being regulated by a single miRNA, that same miRNA is regulating one another disease thus implying a non-direct way (i.e. via a miRNA) of a disease affecting another disease. Hence, if two miRNAs are regulating a single disease, it can be deduced that either the miRNAs are working against each other or in agreement with each other in regulating that particular disease. A significant presence of this motif implies, there are multiple connections of this sort among a diverse set of miRNAs and diseases, which pose a complex networking scenario. A significant amount of 4 node motifs in this network emphasize the earlier observation made above; in that a single miRNA is regulating three diseases, three miRNAs regulating a single disease and two miRNAs regulating a two diseases. This provides a glimpse into the intricate networking of miRNAs and diseases. These results are further corroborated by the findings of MDAN [1], that 64.96% of diseases were *atleast *associated with two miRNAs and about 70% of the miRNAs were associated with two or more diseases. In the 3-node motif and the 4-node motif, the nodes could represent either a miRNA or a disease. However, the edge will always represent an association between a miRNA and a disease. Hence, if a certain node is assumed to be a miRNA, the node lined to it is a disease and vice-versa.

In *DISMIRA *-- a tool developed based on the approach presented in this paper -- upon the input of miRNAs, the top diseases are displayed which participate in maximum number of motifs in the network of entered miRNAs and diseases. Visualization presents an insightful display of the motif structures, thereby providing the research community with a graphical understanding of the nature of association between the miRNAs and diseases.

#### Disease-disease network

**Figure 8:**
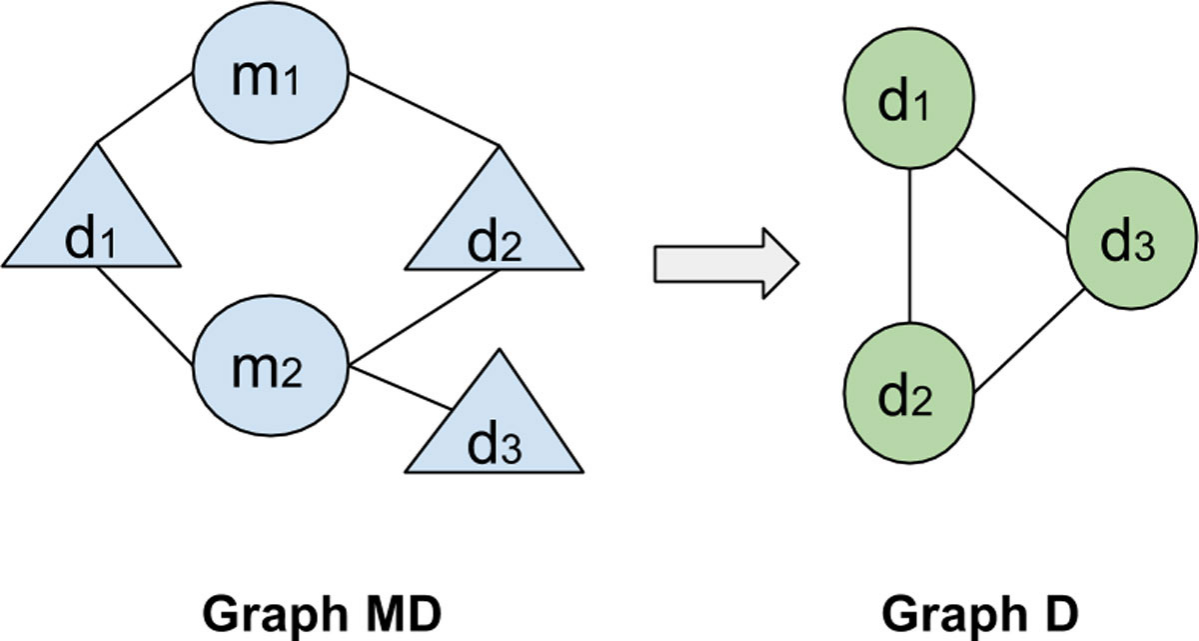
**Example showing the graph transformation from miRNA-disease network to disease-disease network**.

Barabasi, 2007 [32] presents a unique perspective on how social networking in real life spreads pathogens that is revealed in disease network patterns. In order to understand the associations and pattern between the diseases, an exclusive disease-disease network was derived as a projection off the miRNA-disease network. Consider the miRNA-disease network to be graph MD and the disease-disease network to be graph D. An edge between two diseases exists in D if both these diseases are influenced by the same miRNA. This graph transformation is demonstrated in Figure 8.

**Figure 9:**
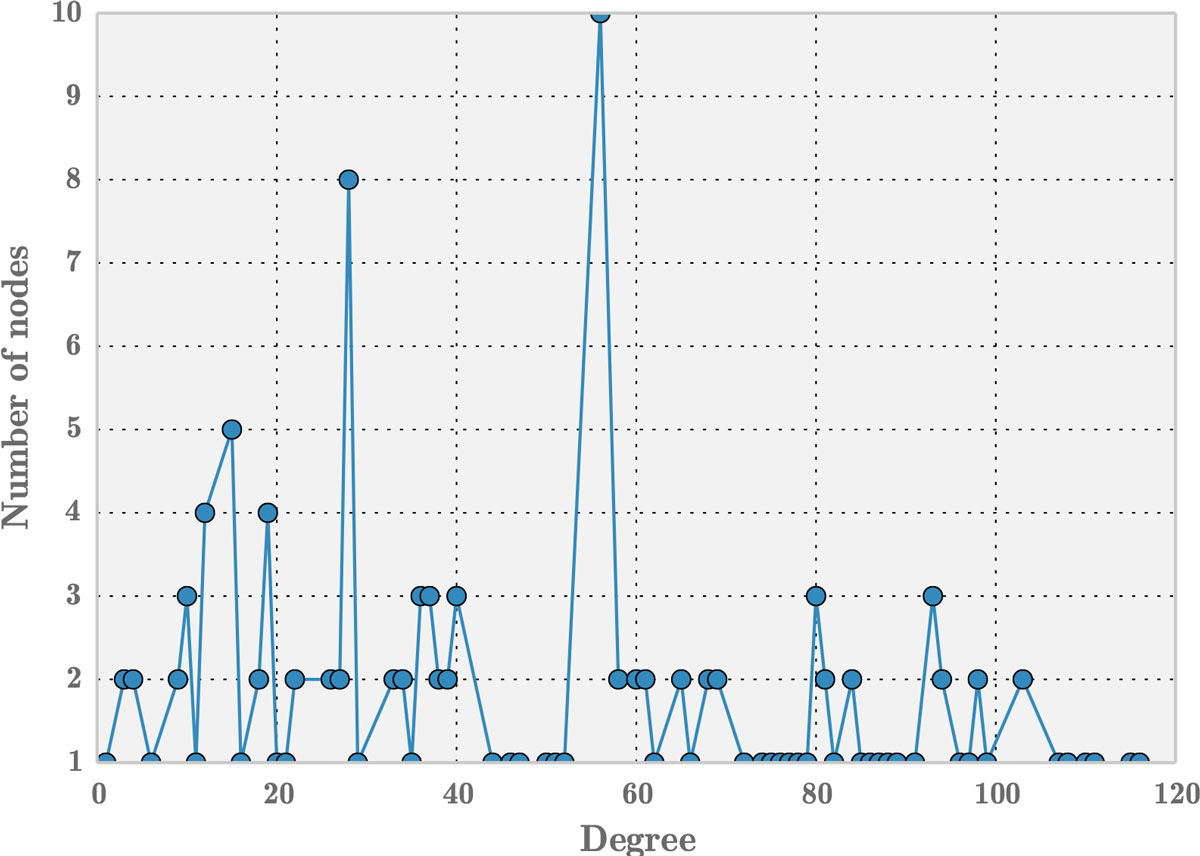
**Degree distribution for disease-disease associations**.

The resulting network has 132 nodes and 3357 edges. To determine the structural properties of disease-disease network, its degree distribution is plotted in Figure 9. Observably, the distribution does not seem to follow power-law distribution which usually indicates scale-free nature of a network. Upon analyses, the same motifs (see Figure 6 and 7) which were observed in miRNA-disease network, were found to be significant in this network. This observation supports the notion that diseases tend to work in tandem with other diseases and in our case via a miRNA passage -- they influence each other.

**Figure 10:**
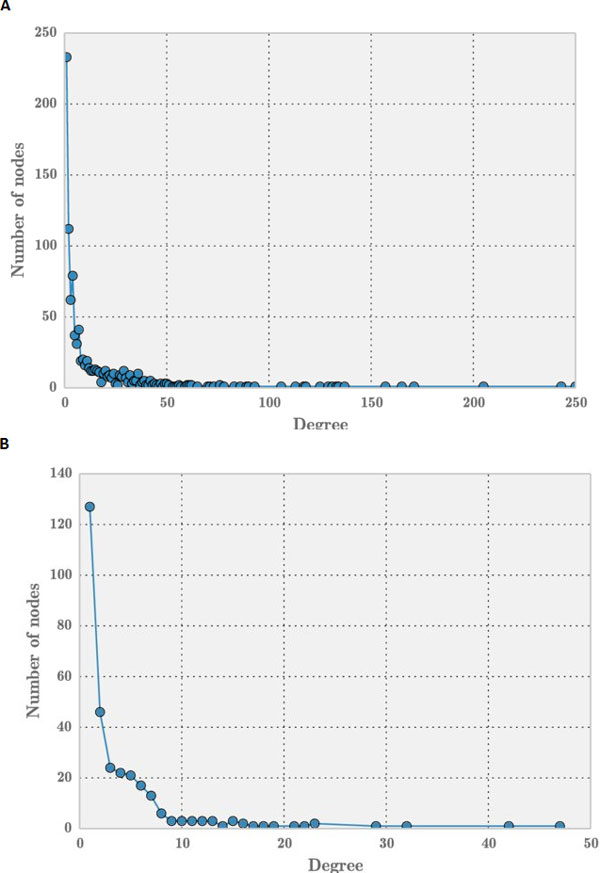
**Degree distribution of miRNA-disease network in (a) HMDD and (b) *mir2Disease***.

These same motifs were observed to be significant in the mirna-disease network of the human microRNA disease database (HMDD) [16] and the *mir2Disease *database [15], hence strengthening the case for these motifs to be vitally important in the available miRNA-disease networks. The HMDD network consisted of 961 nodes (of miRNAs and diseases) and 6448 edges, while the *mir2Disease *database consisted of 309 nodes (miRNAs and diseases) and 637 edges at the time of this paper submission. Also the degree distributions of HMDD and *mir2Disease *follow the nature of scale-free network (see Figure 10 (a) and (b)).

The degree distribution of miRNA-disease network in *miRegulome, HMDD *and *mir2disease *networks in Figures 5 and Figures 10 (a) and (b), respectively reveal a long-tail distribution. Such long-tail degree distributions are referred to as powerlaw distribution wherein few nodes with high degree exist compared to the number of nodes with low degree [33]. It can be understood from these figures that few nodes (i.e miRNAs in our networks) are connected to many neighboring nodes. Thus, exploring the dynamics of nodes can reveal insightful details about miRNA or disease impact.

**Figure 11:**
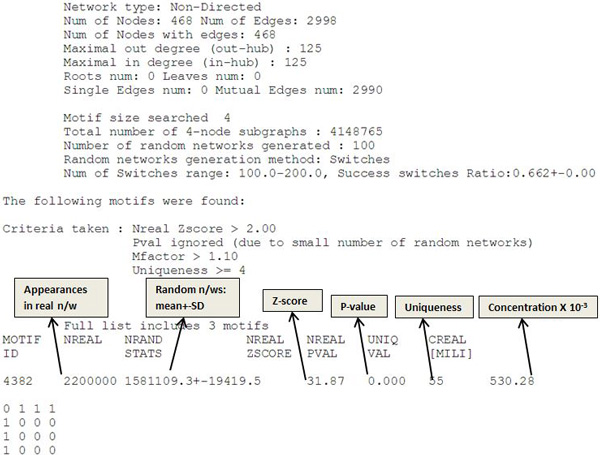
**Summary of results explaining motif identification/significance**.

*mfinder *and *fanmod*, both generate random networks in their process of motif identification. *mfinder *uses 100 random networks and *fanmod *uses 1000 random networks. During this randomizing, 4-node or 3-node sub-graphs are generated among which, the identified motifs have been found to be significant. Figure 11 is an excerpt of the result summary for the significance of 4 node motif in the miRNA- disease network of *miRegulome *by *mfinder*. The explanation has been taken from the manual guide of *mfinder*.

Figure 11 explains the number of occurrences of the 4-node motif in the network, the criteria taken for a motif to be significant, its Z-score, uniqueness and number of random networks generated.

We have incorporated motif-based analysis feature in the tool *DISMIRA*. Upon the input of miRNAs, the tool will display the diseases which have the highest sharing of motif structures with other miRNAs/diseases.

Table 2 shows the diseases and respective motif participation counts for an example input set of miRNAs. Malignant melanoma, Epithelial ovarian cancer (EOC), Breast cancer and Lung cancer are found in thirty seven square motifs.

**Table 2:** Example for disease participation in square motifs with the set of input miRNAs using motif analysis.

Input miRNAs	Disease (participation count in motifs)
hsa-mir-184	Malignant melanoma (37)
hsa-mir-200a	Epithelial ovarian cancer (EOC) (37)
hsa-mir-200b	Breast cancer (37)
hsa-mir-200c	Lung cancer (37)
	Cancer (23)
	Ovarian cancer (OC) (23)
	Serous ovarian cancer (23)
	Hepatocellular carcinoma (HCC) (18)
	Kidney cancer (18)
	Endometriosis (9)
	Non-alcoholic fatty liver disease (9)
	Oral squamous cell carcinoma (OSCC) (8)
	Colorectal cancer (5)

### Network Visualization

**Figure 12:**
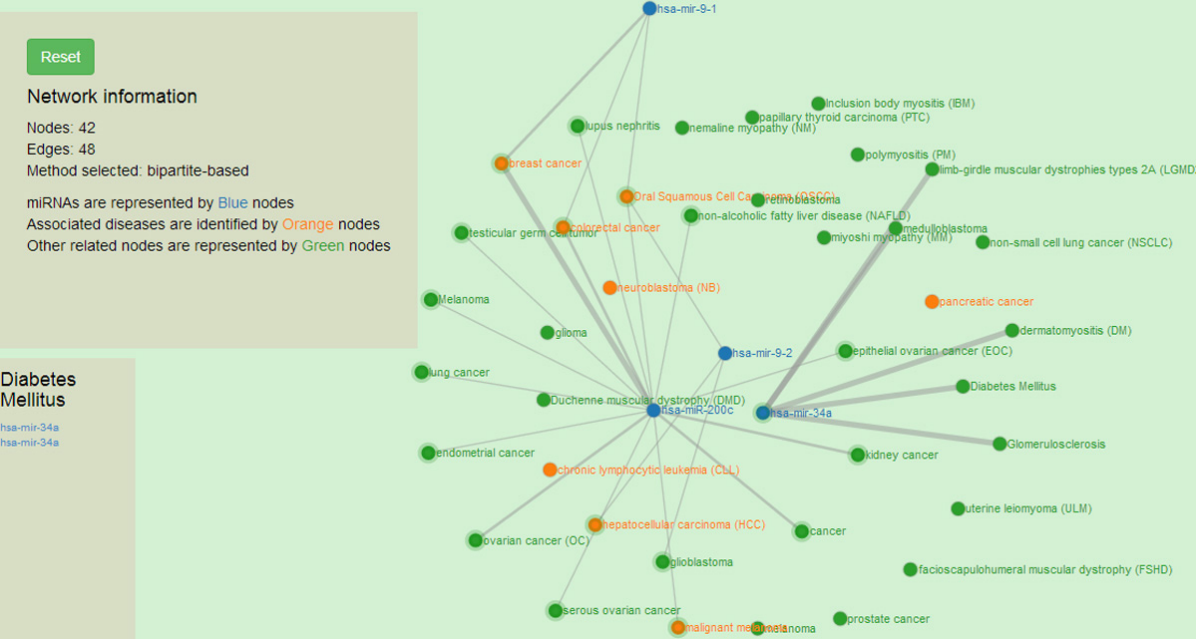
**Visualization of results of maximum weighted matching algorithm**. Blue nodes represent the miRNAs in the network. Green nodes represent diseases associated with the miRNAS. Orange nodes represent the resultant diseases.

**Figure 13:**
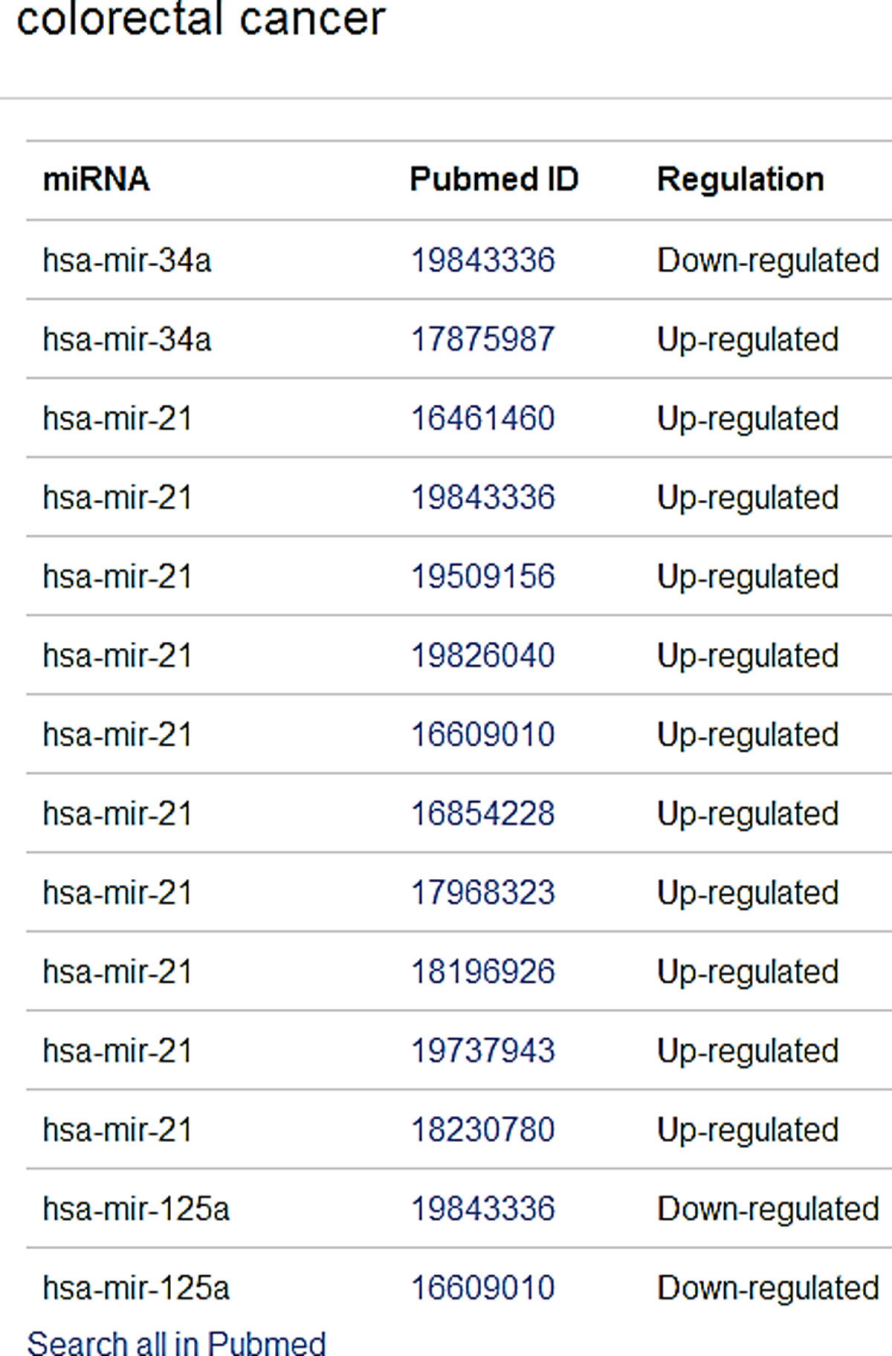
**Regulation details of colorectal cancer**.

The network of miRNAs and diseases can be easily observed in this interactive visualization feature of *DISMIRA*. This insightful perspective into the miRNA-disease associations helps the user in the understanding of the networking of miRNAs and their associated diseases, and also interpreting the the associations among miRNAs and diseases. Maximum weighted matching algorithm and the motif-based analyses are deployed into *DISMIRA *and their results are presented using the interactive visualization. The user can input a set of miRNAs and select either the maximum weighted matching algorithm or the motif-based approach to identify significantly associated diseases, and see their corresponding regulations and PubMed IDs. Upon submitting the input query of miRNAs, the resultant diseases are displayed visually. miRNAs are represented by blue nodes, resultant diseases i.e. the top affected diseases from both the approaches are represented by orange nodes and other associated diseases to the miRNAs are represented by the green nodes (see Figure 12). The resulting miRNA-disease associations are represented using a network visualization in a force-directed layout, meaning placement of miRNAs and diseases are in the most aesthetic way and there is minimal crossing over of edges. This layout makes the understanding of the network very intuitive. Once the results appear, users can zoom-in and zoom-out of the graph for granular level of details such as edge associations, nearby entities and their respective associations etc. Edges between the nodes are disabled by default and are shown upon selecting a specific node. This helps in user-driven network discovery. Upon clicking a miRNA or a disease, its edges are highlighted giving the user, the immediate reach of the entity. Multiple node selections are available to identify nodes of common interest. Interacting with this network visualization of miRNAs and diseases provides helpful insights which are not collected otherwise, such as -- the shortest path from a miRNA/disease to another miRNA/disease, the *k *or *k *+ 1 closest neighbors of a miRNA/diseases and a global perspective of a miRNA or disease's topological placement in the larger picture of this network. Many softwares such as CIDer [34], VisANT [35], InGenuity Pathways Analysis [36], Pathway studio [37] present the network characteristics of biological interactions to facilitate a broader understanding of the systems-level interaction of these complex associations which in various ways govern disease dynamics. However, there are no miRNA-disease interactive visualization tools available for free, as far as we know, at the time of this publication. The visualization tool in this work, generates a user specified network of miRNAs-diseases and allows the user to discover the network with the progression of clicks. The width of the edges i.e. thick and thin, intuitively convey the strength of association between the miRNA-diseases. Furthermore, users can research the top impacted diseases and their subsequent up/down regulation by miRNAs on clicking the disease details and searching them in PubMed literature (see Figure 13). Moreover, the results are displayed intuitively in a 3-way approach; after receiving the list of diseases in the output, the user can click on a certain disease and know *why *the disease is significant (based on the PubMed id count), *where *is it relevant (based on its topological position in the larger picture of miRNA-disease network) and *how *it is impacted (by seeing each miRNA's impact and subsequent regulation towards it). This would assist in thorough investigation. To aid in further detail and completeness for the user, once the network is displayed, all miRNA-disease associations along with their PubMed IDs are provided for download in CSV format. Users use this CSV file in other visualization softwares of their choice too. The visualization is developed using Django framework [38], Python [39] (networkX [40]), JavaScript [41], d3js library [42], bootstrap and HTML with the support of MySQL for back-end database. A snapshot of the visualization is presented in Figure 12. This tool can be accessed for free at: http://bnet.egr.vcu.edu:8080/dismira.

### Case study and utility

Consider the input of miRNAs, hsa-mir-125a, hsa-mir-34a, hsa-mir-21 to *DISMIRA*. Upon choosing the maximum weighted matching (MWM) based model the most impacted diseases are: colorectal cancer, hepatocellular carcinoma (HCC), pancreatic cancer and breast cancer. However, users can select individual miRNAs and observe the association onto other diseases along with the strength of the association. Thicker edges represent high count of PubMed literature supporting the association and regulation (see Figure 12).

Moreover, upon clicking a certain disease, in our example, say 'colorectal cancer' - its subsequent regulation details, PMIDs can be retrieved. In this case, by studying the results further, it can be noted that mir-21 is strongly up-regulated during this disease, whereas mir-125a and mir-34a are being down-regulated (see Figure 13)

**Figure 14:**
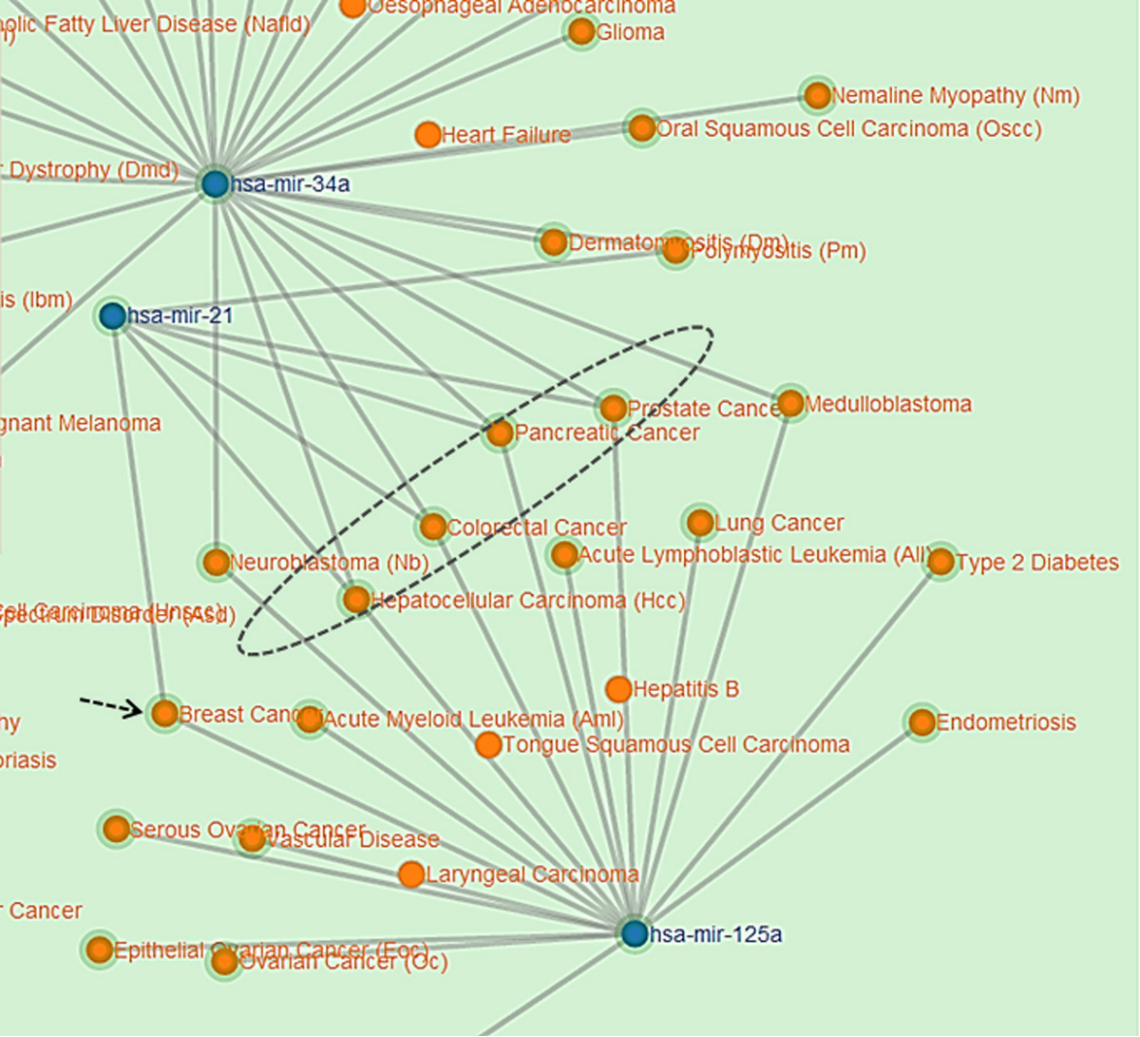
**Motif participation of diseases**.

However, in case of 'pancreatic cancer', mir-21 and mir-125a are both being up- regulated and mir-34a is being down-regulated. The scenarios of multiple miRNAs working together and against each other towards their regulation during a certain disease can be easily observed and studied. Upon selecting the motif-based approach for the same miRNAs, the top diseases are: hepatocellular carcinoma (HCC), colorectal cancer, prostrate cancer and pancreatic cancer with their motif counts of 470, 446, 431 and 317 respectively. These diseases are occurring in most motif structures of 3-node and 4-node. As shown in Figure 14, it is intuitive that these diseases would be in most of the motifs due to their topological placement in the network, prostate cancer, pancreatic cancer and hepatocellular carcinoma are the bordering diseases of three miRNAs' range. Hence, they are most participative in the interaction of several diseases i.e. 3-node and 4-node motifs.

**Figure 15:**
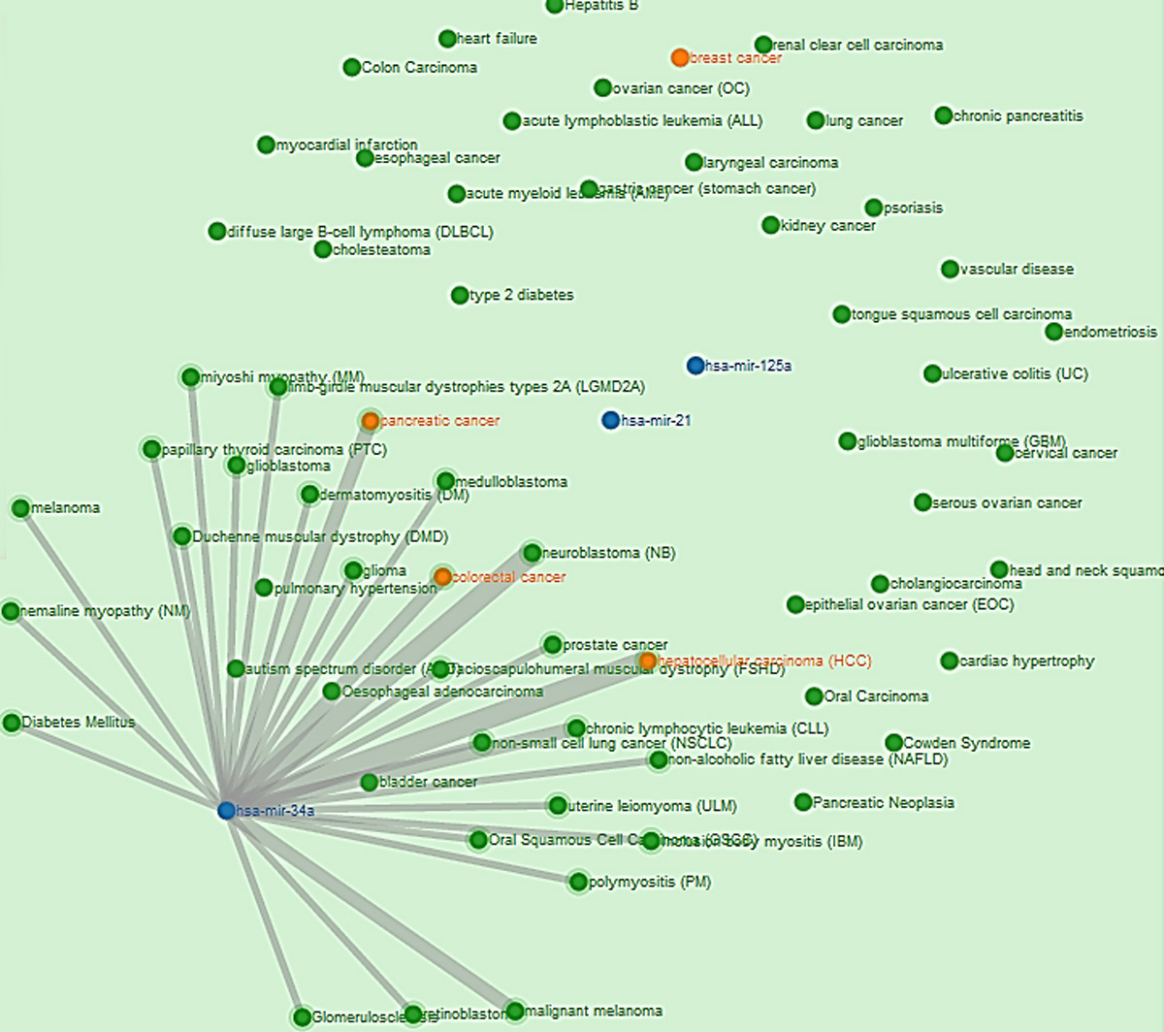
**miRNA's range of influence**.

Whereas, breast cancer (see black dotted arrow in Figure 14) which was one of the resultant diseases in the MWM approach, is listed further below the aforementioned diseases with 265 motifs (observe its placement in the Figure 14) since its being regulated by two miRNAs and thereby less motifs. Upon clicking a certain miRNA, its range of influence can be observed as displayed in Figure 15.

**Figure 16:**
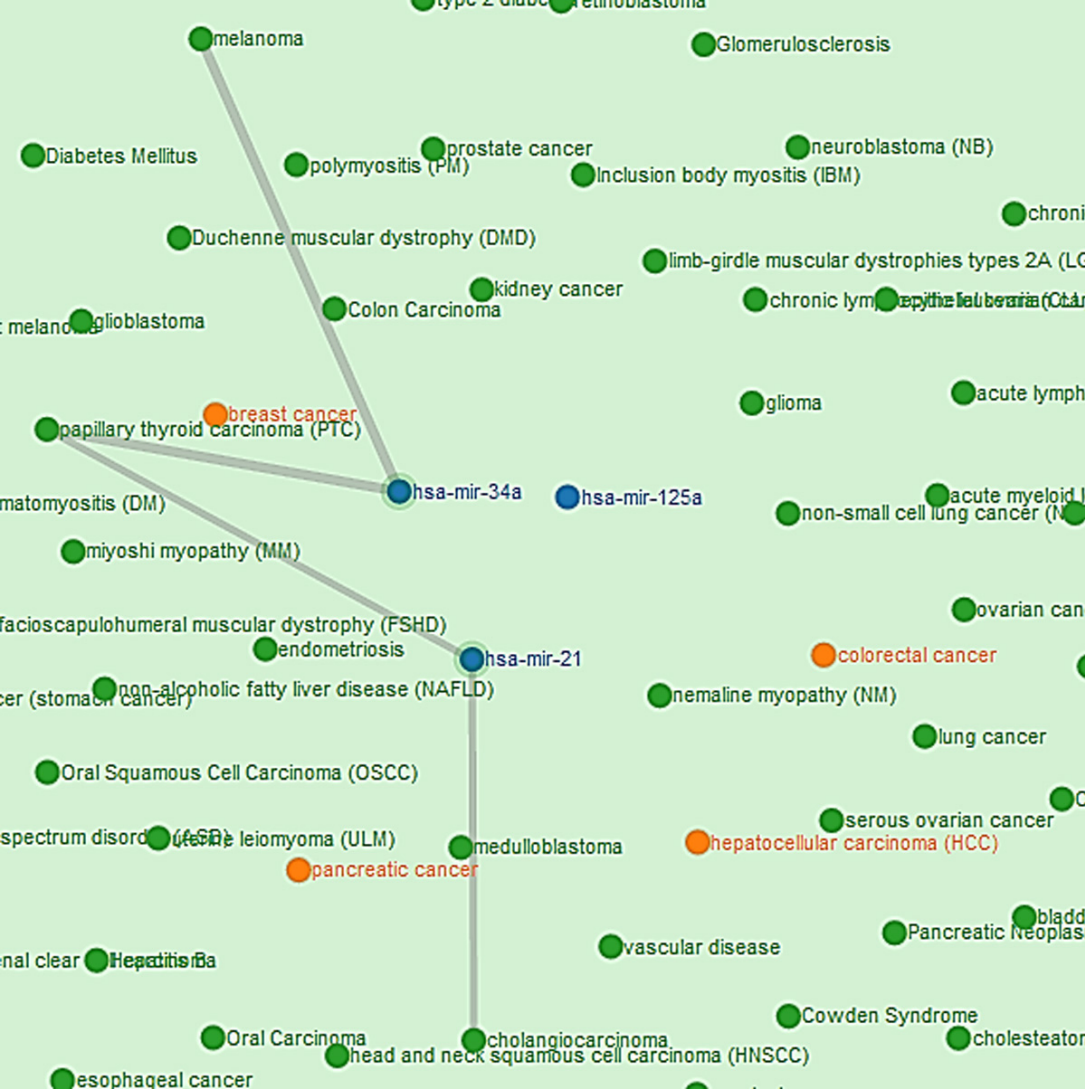
**Paths between diseases**.

Using the visualization, the user can also determine paths or shortest paths to unrelated diseases, for e.g. see Figure 16, the disease cholagiocarcinoma and melanoma seem to be unrelated. However, upon drawing careful egdes, it can be noted that melanoma is three hops away from cholangiocarcinoma, via papillary throid carcinoma (PTC). Upon the activation of the disease PTC, mir-34a and mir-21 are active and thereby the weak possibility of the activation and association of cholangiocarcinoma and melanoma. Similar such paths between diseases of interest can be explored by the user.

It is important to note that, this visualization does not provide strong certainty in predicting or determining the disease-disease interaction, rather merely provides the abstract idea of the reach of the diseases onto each other. However, this tool *does *provide the preliminary overview of the disease-disease interaction network which can be studied adeptly to uncover significant underlying associations.

## Conclusion

Understanding miRNA-disease interactions and their intricate networking has been a goal of biomedical research since many years. In this paper, we present two different network scientific approaches to determine and prioritize disease candidates in a miRNA-disease network based on maximum weighted matching inference model and motif-based analysis. Both these approaches highlight the significant set of diseases based on the queried miRNAs. The visualization aspect provides a topological perspective and a larger understanding of the role and impact of miRNAs and diseases in the network. The results of these approaches, the supporting PubMed IDs and their subsequent regulatory information provide a substantial confidence in the approaches presented in this work. These three features present a novel approach in discovering miRNA-disease ties from diverse viewpoints. This work also allows various possibilities and opportunities of extending this work to introduce miRNA- miRNA ties, expression values between miRNAs and diseases, the role of genes and TFs, and pathways which have already been curated in *miRegulome *(Please see *Introduction*) and incorporate the disease regulation aspect of the miRNA. This research would also introduce algorithms to predict the miRNA-disease association. The database, *miRegulome *would also be enhanced by aggregating further miRNA- disease information, expression values, new associations and attributed from other sources. The established database, maximum weighted inference model, motif-based analyses and the substantial results have paved the way for further work in this domain.

## Competing interests

The authors declare that they have no competing interests.

## Authors' contributions

JJN, BKK and PG conceived, conceptualized and implemented the computational methods. DB, NJ, AB, JJN, BKK and PG developed the miRegulome database. SSA, RTJR, AS, VA and DB validated DISMIRA and provided biological insights. JJN, BKK, PG and DB wrote the manuscript. All authors have read and approved the final manuscript.

## Declarations

Publication costs for this article were funded by NSF grants 1353111 and 1351786.

This article has been published as part of *BMC Genomics *Volume 16 Supplement 5, 2015: Proceedings of the 10th International Conference of the Brazilian Association for Bioinformatics and Computational Biology (X-Meeting 2014). The full contents of the supplement are available online at http://www.biomedcentral.com/bmcgenomics/supplements/16/S5.
